# Comparative Optimism, Self-Superiority, Egocentric Impact Perception and Health Information Seeking: A COVID-19 Study

**DOI:** 10.5334/pb.1139

**Published:** 2022-04-13

**Authors:** Vera Hoorens, Sasha Scambler, Eliane Deschrijver, Neil S. Coulson, Ewen Speed, Koula Asimakopoulou

**Affiliations:** 1 Laboratory for Experimental Social Psychology, KU Leuven, Leuven, BE; 2 Faculty of Dentistry, Oral and Craniofacial Sciences, King’s College, London, UK; 3 Department of Experimental Psychology, University of Ghent, BE; 4 School of Psychology, University of New South Wales, Sydney, NSW, AU; 5 School of Medicine, University of Nottingham, Nottingham, UK; 6 School of Health and Social Care, University of Essex, Colchester, UK

**Keywords:** COVID-19, Beliefs, Comparative Optimism, Self-Superiority, Egocentric Impact Perception, Self-Uniqueness, Perceived Control, Risk Estimation, Risk Factors, Experience

## Abstract

We examined perceived self-other differences (self-uniqueness) in appraisals of one’s risk of an infectious disease (COVID-19), one’s adherence to behavioural precautionary measures against the disease, and the impact of these measures on one’s life. We also examined the relationship of self-uniqueness with information seeking and trust in sources of information about the disease. We administered an online survey to a community sample (N = 8696) of Dutch-speaking individuals, mainly in Belgium and The Netherlands, during the first lockdown (late April-Mid June 2020). As a group, participants reported that they were less likely to get infected or infect others or to suffer severe outcomes than average (unrealistic optimism) and that they adhered better than average to behavioural precautionary measures (illusory superiority). Except for participants below 25, who reported that they were affected more than average by these measures (egocentric impact bias), participants also generally reported that they were less affected than average (allocentric impact bias). Individual differences in self-uniqueness were associated with differences in the number of information sources being used and trust on these sources. Higher comparative optimism for infection, self-superiority, and allocentric impact perception were associated with information being sought from fewer sources; higher self-superiority and egocentric impact perception were associated with lower trust. We discuss implications for health communication.

The COVID-19 crisis has confronted governments with the challenge of promoting behavioural precautions. Even if people find rules generally useful, however, they do not necessarily follow them. Among the phenomena that may limit the effectiveness of health communication targeting the general audience is the belief, arguably held by many, that one differs from one’s peers in important manners (Hoorens, 1993). We call this belief ‘self-uniqueness’. Self-uniqueness may reduce the perceived relevance of available health information for the self, and thus discourage information seeking and reduce trust in potential sources of information. We examined self-uniqueness beliefs in future expectations, self-judgments, and perceptions of the impact of precautionary measures, and their relationship with information seeking and trust in sources of information about how to protect oneself and others against an infectious disease such as COVID-19.

## Self-Uniqueness in Expectations, Self-Judgments, and Impact Appraisals

Comparative optimism is the belief that one’s future will be better than other people’s future (Shepperd et al., 2015; Weinstein, 1980). In studies on comparative optimism, participants typically estimate their likelihood to experience each of a list of given events as compared to the average other or most others (generally specified as ‘of your age and gender’) or estimate absolute likelihoods for themselves and others, with the researchers then calculating self-other differences. A few researchers have examined comparative optimism by having participants judge the severity of negative events for them as compared to others (Hevey & French, 2012) or generate events that may occur in their or other people’s future (Hoorens et al., 2008).

Self-superiority is the belief that one is or acts better than average (Alicke, 1985; Zell et al., 2020). Its measurement typically involves asking participants to judge themselves relative to others on given traits or behaviours or to separately judge the self and others, although some researchers have asked participants to generate examples of their own and others’ behaviours (e.g., Messick et al., 1985).

Finally, egocentric impact perception is the belief that external circumstances and events (e.g., adverse competition circumstances or laws and regulations) affect oneself more than others (Blanton et al., 2001; Chambers et al., 2003b). The hypothetical opposite of egocentric impact perception is allocentric impact perception or the perception that others are more impacted than the self. Egocentric impact perception has been measured by eliciting relative or absolute impact judgments or by inviting people to generate reasons for failures in competitions (Davidai & Gilovich, 2016).

At the individual level, it is virtually impossible to determine the validity of comparative optimism, self-superiority, and egocentric or allocentric impact perception. Some individuals may truly be more likely than average to experience certain outcomes, or less likely to experience other ones. Individuals may also engage in specific actions more or less than average or be more affected by some external circumstance. Yet, at least some individuals must err if most members of a group claim that they are less likely to experience negative events and more likely to experience positive events, that they are or act better, or that they are more (or less) affected by some external circumstance than average (unless the distribution is extremely skewed). For that reason, the general occurrence of comparative optimism has been called unrealistic optimism (Weinstein, 1980), the general occurrence of self-superiority has been called illusory superiority (e.g., Hoorens & Harris, 1998), and the general occurrence of egocentric impact perception has been called the egocentric impact bias (Chambers & Suls, 2007). We borrow these concepts and, in line with them, allocentric impact bias to denote the general occurrence of allocentric impact perception.

Unrealistic optimism has been demonstrated across cultures, ages, and genders, and in various domains (Harris et al., 2008), including health (Hoorens, 1994; Weinstein, 1987). Illusory superiority is also general across ages, genders, cultures, and comparison dimensions (Sedikides et al., 2003, 2007), including health behaviour (Hoorens & Harris, 1998). Fewer studies have focused on egocentric impact perception, but it has similarly been demonstrated across genders, ages, contexts, and types of circumstances (Blanton et al., 2001; Chambers et al., 2003a; Davidai & Gilovich, 2016).

## Differences in Self-Uniqueness and their Behavioural Consequences

Despite the generality of self-uniqueness, research on the two most extensively studied phenomena (comparative optimism, self-superiority) has identified systematic patterns. Unrealistic optimism is greater for events that people perceive as being (vs. not being) under their control, and comparative optimism is greater in individuals who perceived more (vs. less) control (Klein & Helweg-Larsen, 2002). Self-superiority is also greater for traits and behaviours that seem under people’s personal control (vs. uncontrollable; Alicke, 1985) and for morally loaded (vs. non-morally loaded) traits and behaviours (Zell et al., 2020). In the case of unpleasant events, prior experience with events reduces comparative optimism for them. This has been found for, among other events, relational outcomes (e.g., Helweg-Larsen et al., 2011), nature disasters (Burger & Palmer, 1992), and health problems (Larwood, 1978; Weinstein, 1987).

A large literature exists on the consequences of comparative optimism for people’s health and safety behaviour. The research of most direct relevance here is on seeking and using health information. People high on comparative optimism concerning various health issues seek and use less information on these risks (Ahn et al., 2014; Wong, 2012), accumulate less knowledge about them (Davidson & Prkachin, 1997; Lipkus & Klein, 2006; Radcliffe & Klein, 2002), and find the available information less personally relevant (Zlatev et al., 2010).

## Self-Uniqueness Concerning Infectious Disease: COVID-19

### Self-Uniqueness at the Group level

There is reason to expect strong unrealistic optimism and illusory superiority concerning infectious diseases like COVID-19. People arguably perceive getting infected or infecting others as a hazard that is, to a large extent, controllable (Asimakopoulou et al., 2020). During the initial stages of a novel pandemic, moreover, such as during the first wave of COVID-19, very few people have experienced the disease. It is therefore not surprising that a few earlier studies have observed unrealistic optimism concerning COVID-19 during the first half year of the global pandemic (Asimakopoulou et al., 2020; Kuper-Smith et al., 2021; Park et al., 2021). In some studies, men showed greater comparative optimism concerning the disease than women (e.g., Dolinski et al., 2020), although the evidence for these differences is inconsistent (e.g., Asimakopoulou et al., 2020).

However, various aspects of infectious diseases such as COVID-19 are likely to provoke unrealistic optimism to different extents. One reason is the varying degree of control that people arguably perceive over them. Whereas people are likely to perceive infecting others as something that largely depends on how well they adhere to the behavioural precautions, the seriousness of the symptoms that people develop once they are infected arguably seem less controllable (Asimakopoulou et al., 2020). Thus, more unrealistic optimism is to be expected concerning the likelihood of infecting others and getting infected than concerning the seriousness of the illness.

There is also reason to expect illusory superiority. Public health communicators and policy makers have consistently advocated adherence to relatively simple behavioural precautions to stay healthy and save lives. Thus, adherence to the behavioural precautions has been presented as being both under one’s control and as morally loaded. Both are factors that contribute to illusory superiority (Alicke, 1985; Zell et al., 2020).

Finally, the egocentric impact bias may take the form of people’s belief that the precautions imposed by the government affect them more than other people (Sanchez & Gilovich, 2020). However, we are not aware of research showing this phenomenon in the context of COVID-19 or precautionary measures against other infectious disease.

### Individual differences

People show individual differences in the extent to which they consider a disease to be controllable. As noted above, those who perceive more control are likely to show greater comparative optimism than those who perceive less control (Hoorens & Buunk, 1993; Klein & Helweg-Larsen, 2002). Perhaps even more importantly, to the extent that individual differences in self-uniqueness are based on a realistic appraisal of how one stands as compared to others, experience and vulnerability should be associated with different levels of comparative optimism and self-superiority.

#### Experience

The effect of experience on comparative optimism implies that people who have suffered a disease should show lower comparative optimism concerning that disease than people who have not suffered it (Larwood, 1978; Weinstein, 1987). However, the occurrence of that ‘classic’ experience effect was, in the case of our COVID-19 study, by no means self-evident. One reason is that experience with COVID-19 temporarily lowers the likelihood of a (re-)infection. Another reason is that we did our research relatively early in the pandemic. People who by then had survived the disease and had recovered well enough to think of filling out a survey may indeed on average have experienced relatively mild symptoms. Thus, to the extent that comparative optimism is based on a realistic appraisal of one’s relative risk, experience with COVID-19 could enhance rather than reduce comparative optimism for infection and was likely to inflate comparative optimism for severity.

#### Vulnerability

If comparative optimism and self-superiority have a kernel of truth, having or suspecting that one has risk factors should reduce comparative optimism. It should also inspire more precautionary behaviour and thus enhance self-superiority.

At the time of our research, several risk factors had been identified for worse-than-average severity in case of infection. They included old age, male sex, and certain health conditions (e.g., Hashim et al., 2020; Jordan et al., 2020). All these factors quickly became known as factors that affected the severity after in infection, but not (or to a much lesser extent) the likelihood of infection. When people (suspect that they) have risk factors and compare themselves to the average person, their enhanced vulnerability should be associated with lower comparative optimism for severity, but not for infection. It also seems plausible that individuals with a vulnerability adhere to behavioural precautions more meticulously than others do. To the extent that self-superiority also has a kernel of truth, therefore, they should show more self-superiority, which in turn would justify greater comparative optimism for infection.

Concerning the relationship between vulnerability and egocentric impact perception, more meticulously adhering to precautions would imply that these have a greater impact. However, individuals with a vulnerability are likely to already chronically engage in behaviours that in times of pandemics become part of the precautions for society. Moreover, people are likely to appraise the impact of precautions against the background of the impact of the disease itself. That may make the precautions seem much less impactful for those individuals who are (vs. are not) at risk for severe symptoms. We thus predicted that having a vulnerability would be associated with more allocentric (i.e., less egocentric) impact perception.

#### Age and gender

The reasoning behind the predictions described above is that individual differences in self-uniqueness are to some extent realistic. However, a crucial nuance is that the reasoning therefore only holds when people who have risk factors compare themselves to the average person. When they compare themselves with the average person *having the same risk factors*, there is no objective reason for these risk factors to affect self-uniqueness. When people compare themselves to others of the same age and gender, as in many studies on self-uniqueness (e.g., Asimakopoulou et al., 2020; DeGagne & Busseri, 2021), actual differences between age or gender groups cannot explain age and gender effects on self-uniqueness.

## The present research

We tested the hypothesis that self-uniqueness would occur concerning Covid-19. More specifically, we predicted that we would at the group level find unrealistic optimism, illusory superiority, and an egocentric impact bias. We expected greater comparative optimism for infection with, than for severity of COVID-19.

Some predictions of our about individual differences in self-uniqueness assumed that these individual differences are to some extent rooted in actual differences. Thus, we predicted that having risk factors other than being male or being older would be associated with lower comparative optimism for severity, but not for infection, with greater self-superiority, and with lower egocentric impact perception. In contrast, we predicted that experience with COVID-19 would be associated with lower comparative optimism for infection, but not for severity. We did not predict any effects of experience on self-superiority or egocentric impact perception. In contrast, the interpretation of any age and gender effect that might occur would be qualitatively different from vulnerability or experience effects. These effects could not have any real basis because we (as is general practice in self-uniqueness research) asked participants to compare themselves to the average peer of their age and gender.

Finally, we examined the thus far understudied relationship between self-uniqueness concerning COVID-19 and people’s information seeking and evaluation behaviour. Based on earlier research on the relationship between comparative optimism and seeking, accepting, and using health information (e.g., Ahn et al., 2014; Wong, 2012; Zlatev et al., 2010), we expected that the more self-uniqueness individuals showed, the less they would seek information and the less they would trust potential information sources.

## Method

### Participants

The survey was part of a larger, international study on COVID-19 for which we received ethical clearance from King’s College London and confirmation of that clearance as well as privacy clearance through the Social and Societal Ethical Committee of KU Leuven. We report data on the Dutch-speaking sample because of its size, which allowed an analysis of the role of risk factors and, even in a relatively early stage of the pandemic, of personal experience with the disease. At the time of the study (27 April to 11 June 2020), the Dutch-speaking part of Europe (Flemish part of Belgium & The Netherlands) was greatly affected, and a lockdown was in place.

Participants (8696 adults) were invited through researcher contact lists, social media, and news outlets to fill out an online survey.^1^ Demographic and COVID-19-characteristics appear in ***Table 1***. Participants self-categorized in 8 age groups, but we combined the oldest ones (65–74, 75–85, 85+) to achieve roughly equal cell sizes. Our sample size largely exceeds power requirements for all involved analyses. For example, to achieve a 95% chance to observe a small effect (f = .10) in ANOVAs involving age and gender (6 × 2 groups) with an alpha of .05, we needed a sample of N = 1302.

**Table 1:** Demographic characteristics and COVID-19 status.


CHARACTERISTIC	N (8696)	(%)*

Age		

18–24	872	(10.0)

25–34	1496	(17.2)

35–44	1856	(21.3)

45–54	1805	(20.8)

55–64	1729	(19.9)

65-≥ 85	938	(10.8)

Gender		

Women	6030	(69.3)

Men	2633	(30.3)

Other/Neither/Prefer not to say	33	(0.4)

Country		

Belgium	8406	(96.7)

The Netherlands	193	(2.2)

Other*	96	(1.1)

Missing value	1	(<0.1)

Ethnicity		

European	8560	(98.4)

Asian, Black, African or Caribbean	28	(0.3)

Mixed or multiple groups	32	(0.4)

Not in list*	46	(0.5)

Rather not say/Missing value	30	(0.4)

Current experience with symptoms		

No	8438	(97.1)

Yes	256	(2.9)

Missing value	2	(<0.1)

Experience with symptoms		

No	7461	(85.8)

Yes	113	(11.0)

Missing value	282	(3.2)

Experience with COVID-19-testing		

No	8411	(96.7)

Yes, no result	45	(0.5)

Yes, not infected	193	(2.2)

Yes, infected	47	(0.5)

Risk factors	

Absent	5555	(63.9)

Uncertain	1340	(15.4)

Present	1760	(20.2)

Missing value	41	(0.5)


* As reported by participants; among countries of origin were 27 countries with n < 15; a few were regions rather than countries.

### Materials and Procedure

Participants went through an on-screen informed consent procedure where they saw information about the study, advice on what to do in case of symptoms, and information about help lines. They consented by ticking a box, after which the survey started. We describe measures in order of occurrence, except that the section about the impact of the precautions appeared between the general and specific self-superiority items. This was done lest participants took the impact questions as pertaining to only the specific actions in the self-superiority measure.

#### Experience with COVID-19

We asked if participants experienced symptoms (persistent cough, temperature) of what might be COVID-19 (Yes/No), if they had experienced those in the past (Yes, with hospitalization/Yes, without hospitalization/No), and if they had been tested (No/Yes, but I do not have the result yet/Yes, and I was infected/Yes, and I was not infected). We scored participants as having experience if they said yes to at least one item, with the ‘yes’ on the last item having to include the specification that the participant was infected.

#### Comparative optimism

We measured comparative optimism by asking comparative likelihood estimates because COVID-19 was at the time of the study so novel that giving absolute likelihood estimates for themselves and the average other would not have been feasible for most people. More specifically, we asked participants how likely each of 10 events were to happen to them as compared to the average person of their age and gender.

Five events were about getting infected or infecting others: having in the last month infected others, infecting others in the next month, infecting others in the next year, getting (re-)infected in the next month, and getting infected in the next year. Five were about severity in case of (re-)infection: getting symptoms, needing hospitalization, needing to be in an Intensive Care Unit (ICU), needing to be in an ICU and requiring a ventilator or intubation, and fully recovering. The items occurred in a roughly chronological order that would arguably make most sense to participants. First came questions about getting or spreading the infection in the last month and next month, then questions about severity (in order of severity, which corresponds with how the disease may evolved over time in any given patient), followed by full recovery and ending with re(infection) in the next year. Participants answered on 5-point scales from 1 (*Extremely/Much more likely*) to 5 (*Extremely unlikely/Much less likely*).^2^

We subtracted 3 from the likelihood ratings, such that positive scores denoted comparative optimism. A factor analysis (see Supplemental Materials) yielded the expected two factors; we thus created an infection scale and a severity scale (both Cronbach’s α = 0.86).

#### Perceived control

We asked to what extent it was in an individual’s own hand if they got infected and if they infected others (infection items) and if they developed symptoms, needed hospitalization, found themselves in an ICU, found themselves in an ICU and requiring a ventilator or intubation, and fully recover (severity items). Participants answered 1 (*Not at all*), 2 (*A little*), 3 (*Moderately*), 4 (*A lot*), or 5 (*A great deal*). We averaged the two infection items (Cronbach’s α = 0.72), and the 6 severity items (Cronbach’s α = 0.91).

#### Self-superiority

In contrast to likelihood estimates concerning infection with and severity of COVID-19, participants were arguably able to estimate how often they and others had in the recent past performed each behaviour. To measure self-superiority, we therefore asked how often participants and the average other of their age and gender had in the last week shown one recommended behavior (washing one’s hands with water and soap) and four forbidden behaviors: leaving home for fun (other than to exercise once a day), meeting a friend or relative not living in one’s household, visiting other people at home, running an errand that is not necessary. We also included two behaviors that in principle were allowed but that people were requested to engage in only when they were strictly necessary, and not if they experienced any COVID-19 symptom: shopping for groceries and going to a pharmacy or a physician. Participants answered with 1 (*Never*), 2 (*Once*), 3 (*On 2–3 days*), 4 (*On 4–5 days*), 5 (*Almost daily*), 6 (*Daily*), 7 (*Several times per day*). We recoded ratings into a scale from 0 to 6, such that 0 corresponded to ‘never’. We reverse-coded the hand hygiene items, subtracted the rating for the average other from the rating for the self, and calculated a mean self-superiority score across the seven behaviours (Cronbach’s α = 0.66).

Because we also wished to include a self-superiority measure that was methodologically comparable to the comparative optimism measures, we also used a directly comparative approach to measure participants’ general appraisal of how well they adhered to the behavioural precautions. Participants indicated how well, as compared to the average person of their age and gender, they had in the last month complied and would in the next month comply with social distancing rules (defined as keeping one’s distance from others and staying at home). They did so on a 5-point scale from 1 (*Much better*) to 5 (*Much worse*). We reverse-coded ratings and subtracted 3 to create self-superiority scores (positive values denoting self-superiority). Lest participants interpreted these questions as meaning ‘apart from the behaviours mentioned in the frequency items’, the general questions preceded the items about specific behaviours.

#### Egocentric impact perception

We measured egocentric impact perception through directly comparative questions because participants would arguably find it next to impossible to estimate how much the precautions affected other people. Moreover, we wished to avoid response shift, with labels meaning different levels in different groups (e.g., Ogden & Lo, 2012). Again, we measured a general appraisal and more specific appraisals.

Participants indicated how complicated it was for them to comply with social distancing rules as compared to the average person of their age and gender, on a 5-point scale from 1 (*Much more complicated*) to 5 (*Much less complicated*). We recoded their responses such that positive scores indicated greater impact on the self (egocentric impact perception) and negative scores less impact on the self (allocentric impact perception).

We also asked how much the rules adversely affected aspects of their life, as compared to the average person of their age and gender: daily routine, mood, income, hobbies, contact with individuals from outside the household, and contact with household members. Participants answered on a 5-point scale from 1 (*Much less*) to 5 (*Much more*). We calculated a mean egocentric impact score (Cronbach’s α = 0.74).

#### Information seeking and trust in sources of information

To avoid demand effects, social desirability, and commonality in methods, we did not measure information seeking by asking participants to self-judge how eagerly they sought information. Instead, we asked them to indicate which information sources they used, and from their responses derived an index that reflected how broadly their information search was. This index served as a proxy for the extent to which participants sought information. More specifically, we asked participants if they obtained information about COVID-19 from the following (non-exclusive) categories: friends, relatives, television, newspapers, the government, internet, social media, and ‘other’. Participants were asked to tick all that applied. We counted the sources and thus obtained an information seeking score from 0 to 8. The higher this score, the more broadly participants arguably sought for information about COVID-19.

In addition, we asked how well participants trusted each of the listed sources to give accurate information about COVID-19. They answered this question, per source, on a 5-point scale from 1 (*A great deal*) to 5 (*Not at all*). After recoding, higher scores denoted greater trust. We calculated a score for trust in information sources by averaging over sources (Cronbach’s α = 0.71).

#### Risk factors

Participants indicated if they had a health condition that made one more vulnerable to COVID-19: “Yes”, “I am not sure”, “No”. We then showed a list of risk factors (see Supplemental Materials) and asked participants to tick those that applied. From their responses, we derived a vulnerability score that took the values low, uncertain, or high. Participants were assigned the value ‘low’ if they had picked “No”. They were assigned the value ‘uncertain’ if they had picked “I am not sure” or had picked “Yes” but without selecting any specific factor, as we interpreted the latter as indicating a feeling of being at risk without being able to specify why. Participants were assigned the value ‘high’ if they had picked “Yes” and had selected at least one risk factor other than being over 70 years old. We did not count being over 70 years old because the measure of comparative optimism required participants to compare themselves with people of their age (but not with people with the same status on other risk factors), such that ‘age’ did not objectively enhance people’s relative likelihood to suffer more in case of an infection.

## Data Sharing Statement

The data file, codebook, syntax, output, and research materials are available on OSF (*https://osf.io/q3ugn/*).

## Results

We report all significances, but do not follow up on effect sizes < .005 to avoid over-interpretation. We round effect sizes .005–.009 to 0.01. Confidence intervals (CI) are 95% intervals of differences, and *d*s are Cohen’s *d*s. We tested contrasts for binary variables with t-tests, and for others with Tukey tests. The overall means are in ***Table 2***.

**Table 2:** Correlations between comparative optimism (CO), self-superiority, egocentric impact perception, perceived control, and information use and trust.


	*M*	*SD*	2	3	4	5	6	7	8

1 CO infection	0.58	0.72	.20**	.21**	.16**	.20**	–.27**	–.05**	.03*

2 CO severity	0.26	0.73		–.01	.11**	–.11**	.05**	.00	.01

3 Control infection	3.32	0.85			.16**	.21**	–.17**	.00	.06*

4 Control severity	1.48	0.75				.10**	–.04**	–.03**	–.01

5 Self–superiority^#^							–.21**	–.02*	–.05**

6 Egocentric impact^#^								.06**	–.04**

7 Information seeking	3.94	1.52							.23**

8 Source trust	3.38	0.73							


^#^ Based on standardized scores on general and specific items * p < .05; ** p < .005.

### Unrealistic Optimism, Illusory Superiority, and Egocentric Impact Bias

We predicted that, at the group level, we would find unrealistic optimism, illusory superiority, and an egocentric impact bias.

We tested the occurrence of unrealistic optimism through one-sample t-tests on the mean comparative optimism scores. These scores were higher than zero for infection, *t*(8676) = 74.76, *p* < .001, *d* = 0.80, CI [0.56, 0.59], and severity, *t*(8659) = 32.89, *p* < .001, *d* = 0.35, CI [0.24, 0.27]. A paired-samples t-test revealed stronger unrealistic optimism for infection than for severity: *t*(8657) = 32.31, *p* < .001, *d* = .35, CI [0.30,0.34]. Perceived control was also higher for infection than for severity, *t*(8676) = 163.72, *p* < .001, *d* = 1.76, CI [1.81, 1.86]. Participants considered infection moderately to ‘a lot’ controllable, and severity not all or just a little controllable. To examine if the difference in perceived control explained (part of) the difference in comparative optimism, we calculated difference scores for control (over infection minus over severity) and ran a repeated measures ANOVA on the comparative optimism scores with the difference score as a covariate. Supporting the view that differences in perceived control accounted for part of the greater comparative optimism for infection than for severity, covariate interacted with aspect, *F*(1, 8643) = 110.87, *p* < .001, *η_p_^2^* = .01. The main effect of aspect was still significant, *F*(1, 8643) = 45.95, *p* < .001, *η_p_^2^* = .01, but was much smaller than in a similar analysis without the covariate, *F*(1, 8657) = 1033.18, *p* < .001, *η_p_^2^* = .11. As predicted, we found unrealistic optimism for infection and severity; also as predicted, it was stronger for the aspect of COVID-19 that participants perceived as being relatively controllable (infection) than for the aspect that they perceived as being relatively uncontrollable (severity).

We tested the occurrence of illusory superiority in two manners. One consisted of one-sample t-tests on the general self-superiority ratings for the past and the future. These scores were higher than zero for both past adherence (*M* = 1.02, *SD* = 0.84), *t*(8689) = 113.23, *p* < .001, *d* = 1.22, CI [1.00,1.04], and future adherence (*M* = 0.75, *SD* = 0.91), *t*(8684) = 77.29, *p* < .001, *d* = 0.83, CI [0.73, 0.78]. However, we unexpectedly found that illusory superiority was lower for the next than for the past month, as shown by a paired samples t-test, *t*(8681) = 34.88, *p* < .001, *d* = 0.83, CI [0.25, 0.28].

The other test involved a one-sample t-test on the average self-superiority scores across the specific behaviours for which participants had given frequency estimates. The mean score was higher than zero (*M* = 0.60, *SD* = 0.57), *t*(8493) = 97.32, *p* < .001, *d* = 1.06, CI [0.59, 0.61]. As predicted, we thus found illusory superiority, both in general appraisals of one’s relative adherence to the precautionary measures and in estimates of the frequency of specific behaviors.

We also tested the occurrence of an egocentric impact bias in two manners. First, we conducted a one-sample t-test on the general rating of the difficulty of adhering to the precautions. The mean score was lower than zero (*M* = –0.27, *SD* = 1.12), *t*(8650) = 22.39, *p* < .001, *d* = –.24, CI [–0.29, –0.25]. Second, we conducted a one-sample t-test on the mean impact ratings for specific life domains. Again, the mean score was lower than zero *M* = –0.07, *SD* = 0.74), *t*(8679) = 8.42, *p* < .001, *d* = –0.09, CI [–0.08, 0.05]. Contrary to prediction, we thus observed an allocentric rather than an egocentric impact bias.

The Supplemental Materials include tests of self-uniqueness per event, behaviour, and life domain. Comparative optimism occurred for all events, and illusory superiority for all behaviours. However, the life domains differed with respect to whether they provoked an egocentric impact bias, an allocentric impact bias, or no bias at all.

### Differences in Comparative Optimism, Self-Superiority, and Impact Perception

Participants who felt that COVID-19 was more under one’s personal control showed more comparative optimism than participants who felt that COVID-19 was less under one’s personal control (see the positive correlations in ***Table 2***).

#### Experience and vulnerability

We tested the predictions that being vulnerable due to having risk factors other than being male or old would be associated with lower comparative optimism for severity, greater self-superiority, and lower egocentric impact perception, whereas experience with COVID-19 would be associated with lower comparative optimism for infection. We therefore subjected self-uniqueness scores to ANOVAs with experience and vulnerability as between-subjects variables. In the case of comparative optimism scores, we also included aspect as a within-subjects variable. In the case of general self-superiority scores, we included time (self-superiority for the past month, for the next month) as a within-subjects variable.

##### Effects on comparative optimism

Besides the already described effect of aspect, we found a main effect of vulnerability, *F*(2, 8490) = 106.55, *p* < .001, *η_p_^2^* = .02. Most importantly, we found the predicted interactions of aspect by vulnerability, *F*(1, 8490) = 390.01, *p* < .001, *η_p_^2^* = .08, and aspect by experience, *F*(1,8490) = 92.50, *p* < .001, *η_p_^2^* = .01.

As predicted, participants with experience with COVID-19 showed lower comparative optimism for infection (*M* = 0.45; *SD* = 0.75) than participants without (*M* = 0.60; *SD* = 0.71), *t*(1587.89) = 6.65, *p* < .001, *d* = 0.21, CI [0.11, 0.20]. In contrast, they showed higher comparative optimism for severity (*M* = 0.33; *SD* = 0.74) than participants without experience (*M* = 0.25; *SD* = 0.73), *t*(1617.31) = 3.66, *p* < .001, *d* = –.12, CI [–0.13, –0.04].

Also as predicted, participants high on vulnerability showed lower comparative optimism for severity (in fact, they showed comparative pessimism; *M* = –0.29; *SD* = 0.82) than ‘uncertain’ participants (*M* = 0.14; *SD* = 0.65), *p* < .001, *d* = –.68, CI [0.39, 0.51], and these showed lower comparative optimism than participants low on vulnerability (*M* = 0.46; *SD* = 0.61), *p* < .001, *d* = –.49, CI [0.28, 0.37]. In contrast, participants high on vulnerability showed greater comparative optimism for infection (*M* = 0.71; *SD* = 0.73) than ‘uncertain’ participants (*M* = 0.59; *SD* = 0.72), *p* = .012, *d* = 0.09, CI [–0.11, –0.01], and these participants showed greater comparative optimism than in participants low on vulnerability (*M* = 0.53; *SD* = 0.71), *p* < .001, *d* = 0.16, CI [–0.17, –0.05].

##### Effects on self-superiority

Besides the effect of time, we found a small Time × Experience interaction *F*(1,8515) = 5.91, *p* = .015, *η_p_^2^* < .005. Most importantly, we found the predicted effect of vulnerability, *F*(2, 8515) = 48.99, *p* < .001, *η_p_^2^* = .01. Participants high on vulnerability showed more self-superiority (*M* = 1.12, *SD* = 0.79), than ‘uncertain’ participants (*M* = 0.96, *SD* = 0.79), and these showed more self-superiority than participants low on vulnerability (*M* = 0.80, *SD* = 0.79), *p*s < .001. However, the mean self-superiority score was above zero in all groups, *t*s > 75.35, *p*s < .001, *d*s ≥ 0.20.

The ANOVA on mean self-superiority scores yielded a small effect of experience, *F*(1,8337) = 4.77, *p* = .029, *η_p_^2^* < .005, and the predicted effect of vulnerability, *F*(2,8337) = 33.78, *p* < .001, *η_p_^2^* = .01. Participants high on vulnerability showed more self-superiority (*M* = 0.70, *SD* = 0.60) than ‘uncertain’ participants (*M* = 0.65, *SD* = 0.60), *p* = .022, *d* = 0.09, and both groups showed more self-superiority than participants low on vulnerability (*M* = 0.55, *SD* = 0.54), *p*s < .001, *d*s ≥ 0.18.

As noted in the introduction, greater self-superiority among participants high on vulnerability might be due to a realistic appraisal of their own adherence to the precautions being better than other individuals’ adherence. To examine if that was indeed the case, we did a follow-up analysis on raw frequency scores with target (self, other) as a within-subjects variable and vulnerability as a between-subjects variable. We found effects of target, *F*(1,8362) = 7406.50, *p* < .001, *η_p_^2^* = .47, and Target × Vulnerability, *F*(2, 8362) = 50.64, *p* < .001, *η_p_^2^* = .01. Participants high on vulnerability indeed felt that they had violated the rules less often (*M* = 0.50, *SD* = 0.37) than ‘uncertain’ participants (*M* = 0.54, *SD* = 0.41), *p* = .007, or than participants low on vulnerability (*M* = 0.57, *SD* = 0.39), *p* < .001. These groups also differed, *p* = .022. However, participants high on vulnerability also felt that others had violated the rules more often (*M* = 1.20, *SD* = 0.58) than ‘uncertain’ participants did (*M* = 1.20, *SD* = 0.55), *p* < .001, or than participants low on vulnerability did (*M* = 1.13, *SD* = 0.50), *p*s < .001. These groups did not differ, *p* = .988. Thus, the finding that participants high on vulnerability showed more self-superiority than participants low on vulnerability was not only driven by their (perhaps accurate) perception that they adhered better to the precautions, but also by their perception that other people adhered more poorly to them.

##### Effects on egocentric/allocentric impact perception

Besides a small effect of experience, *F*(1,8484) = 19.38, *p* < .001, *η_p_^2^* < .005, we found the predicted effect of vulnerability, *F*(2,8484) = 19.54, *p* < .001, *η_p_^2^* = .01. Participants high on vulnerability showed the greatest allocentric impact perception (*M* = –0.47, *SD* = 1.15), followed by ‘uncertain’ participants (*M* = –0.35, *SD* = 1.11), and participants low on vulnerability (*M* = –0.19, *SD* = 1.10), pairwise comparisons significant at *p* < .013. An ANOVA on the relative impact scores for specific domains did not yield any effect, *F*s(1, 8514) ≤ 3.77, *p*s ≥ .052.

#### Age and gender differences in self-uniqueness

We subjected self-uniqueness scores to ANOVAs with age and gender as between-subjects variables. In the case of comparative optimism scores, we again included aspect as a within-subjects variable. In the case of general self-superiority scores, we included time as a within-subjects variable. Because participants were asked to compare themselves to the average other of their age and gender, differences between age and gender groups may meaningfully be described as differences in *unrealistic* optimism, *illusory* self-superiority, and egocentric (or allocentric) impact *bias*.

##### Effects on unrealistic optimism

**Figure 1:**
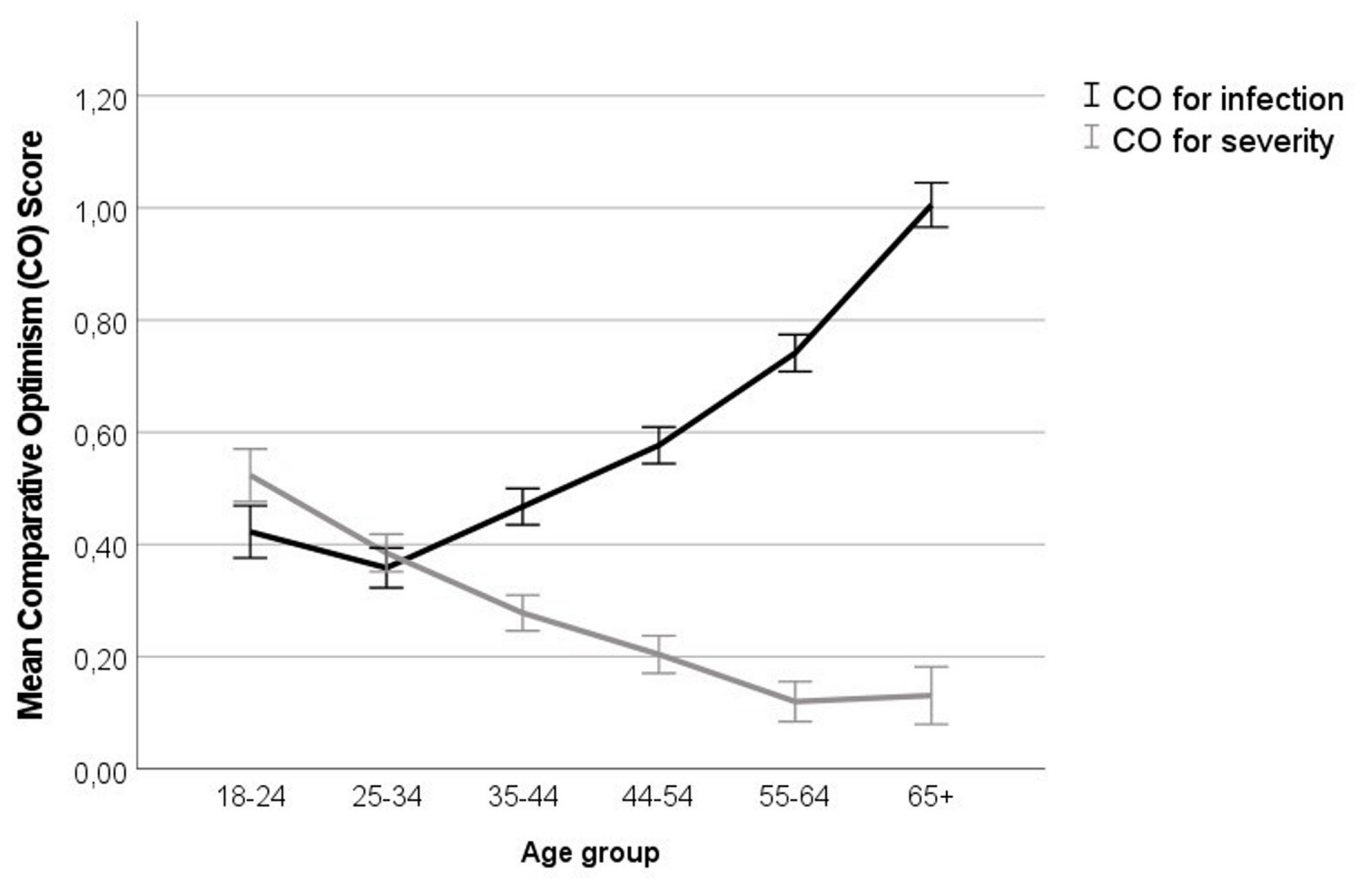
Mean scores for infection-related and outcome-related comparative optimism (CO), as a function of age. Positive scores denote comparative optimism. Error bars denote 95% confidence intervals.

Besides the already mentioned effect of aspect, we found a main effect of age, *F*(5,8613) = 14.05, *p* < .001, *η_p_^2^* = .01; and a two-way interaction of aspect by age, *F*(1,8613) = 177.84, *p* < .001, *η_p_^2^* = .09. As shown in ***Figure 1***, older (vs. younger) participants showed lower unrealistic optimism for severity but more for infection. There were small effects of gender, *F*(1,8613) = 38.42, *p* < .001, and age by gender, *F*(5,8613) = 3.01, *p* = .010, both *η_p_^2^* < .005.

##### Effects on illusory superiority

**Figure 2:**
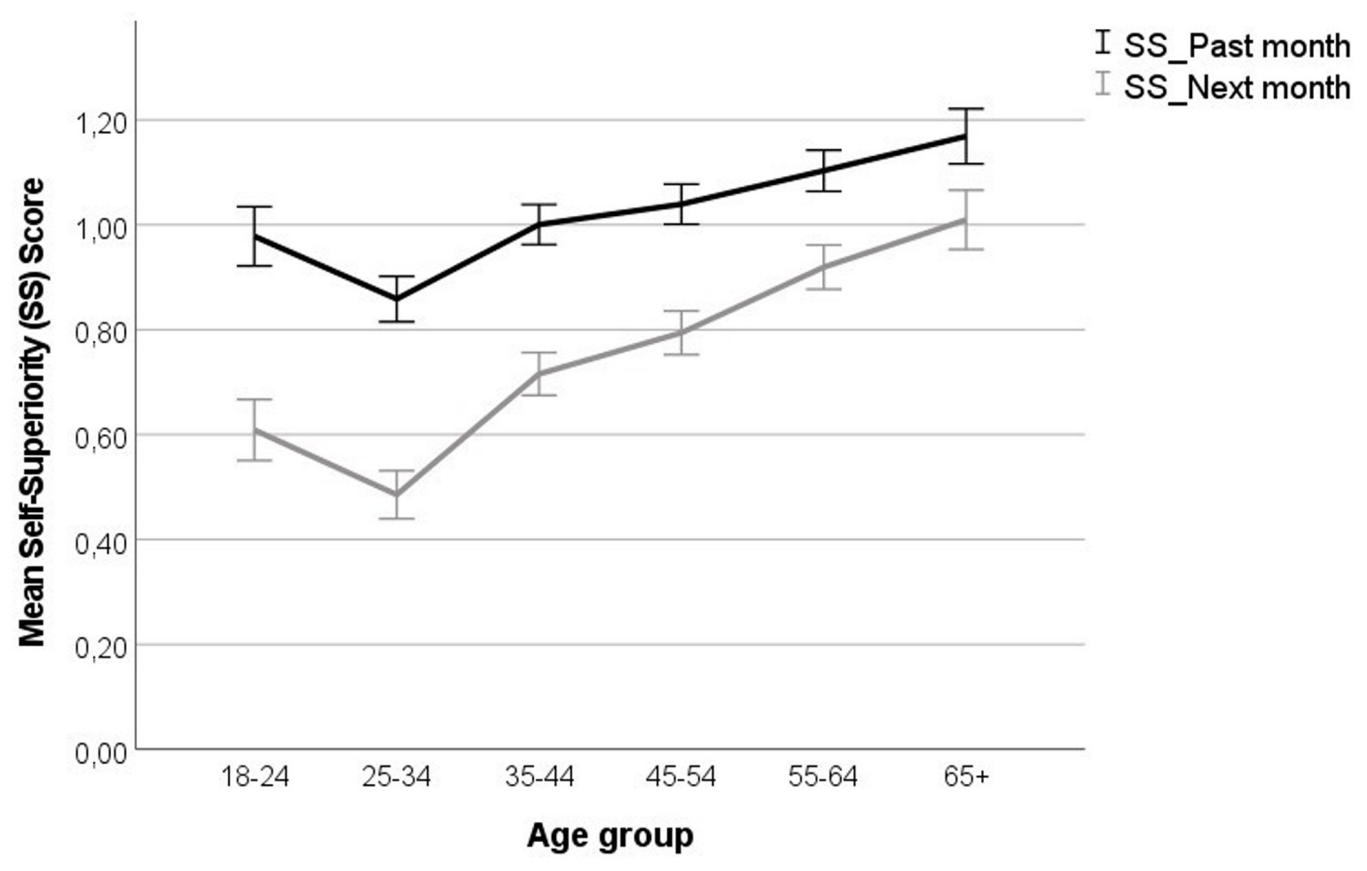
Mean self-superiority (ss) scores for adherence to the precautionary rules in the last month and the next month, per age group. Positive scores denote self-superiority. Error bars denote 95% Confidence Intervals.

Besides the effect of time, the ANOVA on the general self-superiority score yielded a small Time × Age × Gender interaction, *F*(1,8637) = 4.20, *p* = .001, *η_p_^2^* < .01. More importantly, it yielded a main effect of age, *F*(5,8637) = 36.65, *p* < .001, *η_p_^2^* = .02, and a Time × Age interaction, *F*(1,8637) = 13.61, *p* < .001, *η_p_^2^* = .01. As ***Figure 2*** shows, illusory superiority was greater in older (vs. younger) participants, and the past-next month difference in illusory superiority was smaller in older (vs. younger) participants. The ANOVA on the mean superiority score across specific behaviours only yielded a small effect of age, *F*(1,8449) = 6.54, *p* < .001, *η_p_^2^* < .005; other *F*s < 2.44, *p*s ≥ .118.

##### Effects on egocentric/allocentric impact bias

The ANOVA on the mean relative impact scores yielded effects of age, *F*(5,8607) = 52.23, *p* < .001, *η_p_^2^* = .03, and gender, *F*(1,8607) = 16.82, *p* < .001, *η_p_^2^* < .005. The allocentric impact bias was greater in older than in younger participants (< 25: *M* = –0.12, *SD* = 0.92; 25–34: *M* = –0.12, *SD* = 0.93; 35–44: *M* = –0.30, *SD* = 0.92; 45–54: *M* = –0.42, *SD* = 0.94; 55–64: *M* = –0.55, *SD* = 0.94; ≥ 65: *M* = –0.69, *SD =* 0.92); except between the youngest groups, all pairwise differences were significant, *p*s < .01. However, the allocentric impact bias occurred at all ages, one-sample t-tests (test value = 0): *t*s ≥ 3.99, *p*s < .001.

**Figure 3:**
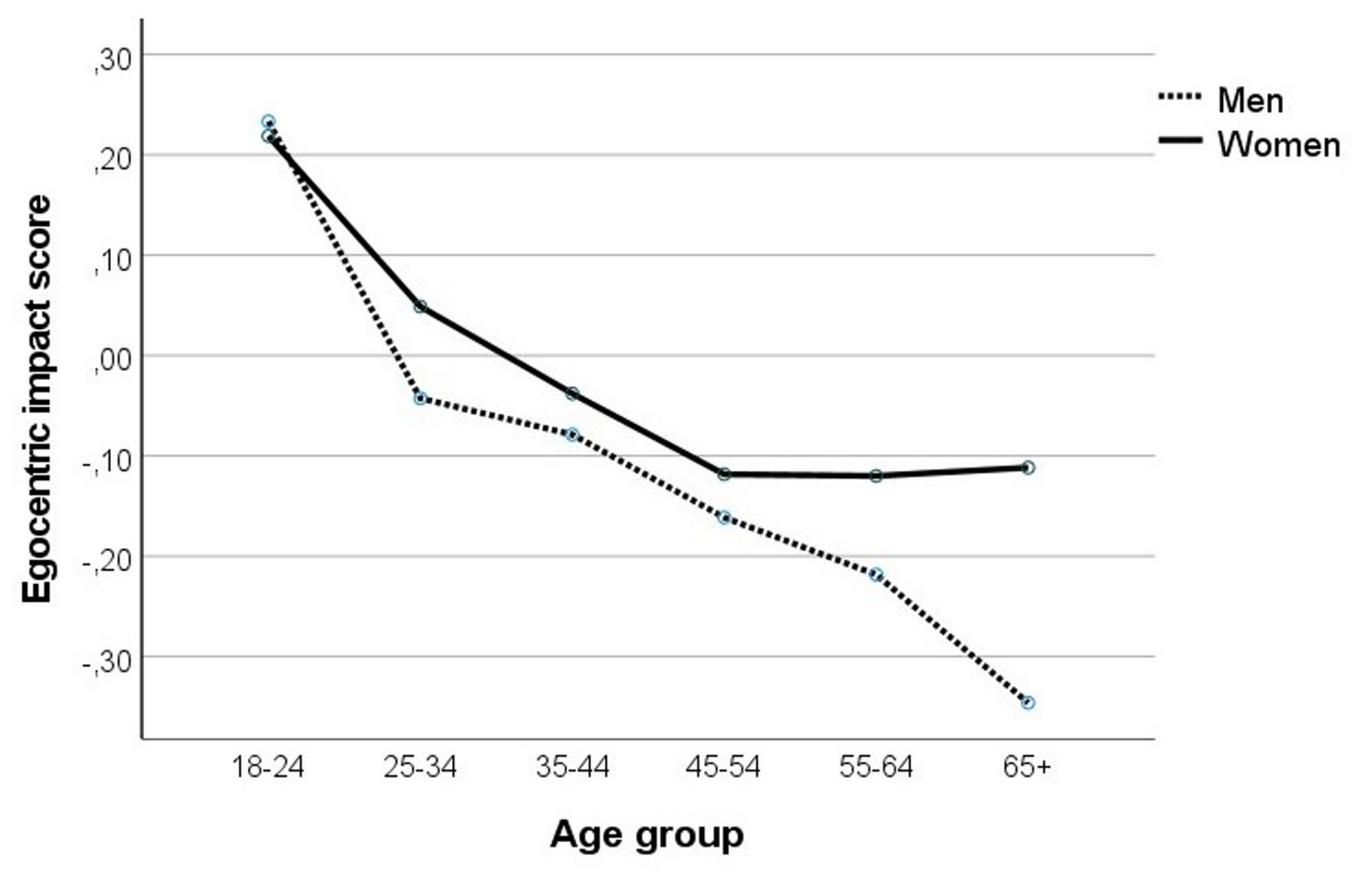
Average egocentric impact score across specific life domains as a function of age and gender. Positive values indicate greater impact, and negative scores lower impact on the self than on others.

The ANOVA on the mean relative impact scores for specific domains yielded small effects of gender, *F*(1,8635) = 19.78, *p* < .001, *η_p_^2^* < .005, and age by gender, *F*(5,8635) = 3.04, *p* = .010, *η_p_^2^* < .005. More importantly, it yielded a main effect of age, *F*(5,8635) = 37.10, *p* < .001, *η_p_^2^* = .02. As shown in ***Figure 3***, the older participants were, the greater their allocentric impact bias.

Participants under 25 years old were the only ones who showed a significant egocentric impact bias (*M* = 0.22, *SD* = 0.65), *t*(817) = 10.03, *p* < .001, *d* = .34, CI [0.18, 0.26]. Among those of 25–34 years old the egocentric impact bias was not significant (*M* = 0.03, *SD* = 0.71), *t*(1494) = 1.63, *p* = .104, *d* = .04, CI [–0.01, 0.07]. All older age groups showed a significant allocentric impact bias: 35–44 years olds (*M* = –0.05, *SD* = 0.73), *t*(1854) = 2.74, *p* = .006, *d* = –.06, CI [–0.08, –0.01]; 45–54 years olds (*M* = –0.13, *SD* = 0.75), *t*(1802) = 7.52, *p* < .001, *d* = –.18, CI [–0.17, –0.10], 55–64 years olds (*M* = –0.16, *SD* = 0.74), *t*(1725) = 8.92, *p* < .001, *d* = –.22, CI [–0.19, –0.12] and above all 65+ years olds (*M* = –0.23, *SD* = 0.75), *t*(928) = 9.39, *p* < .001, *d* = –.31, CI [–0.28, –0.18]. All pairwise comparisons between age groups were significant at *p* ≤ .031, except for the difference between the 45–54 and 55–64 years old, and between the 55–64 and 65+ years old.

### Association between self-uniqueness, information seeking, and trust in sources

We examined how self-uniqueness was related to participants’ information seeking and trust in information sources. For clarity, we calculated overall self-superiority and egocentric impact perception scores. The overall self-superiority score averaged standardized scores on the general ratings (after having pooled general ratings for the last and the next month, *r* = .67) and mean self-superiority scores for specific behaviours. The overall egocentric impact score averaged standardized scores on the general item and on the specific scores across specific domains.

Comparative optimism for infection and self-superiority were associated with lower information seeking; self-superiority and egocentric impact perception were associated with lower trust. In contrast, egocentric impact perception was positively correlated with information seeking and comparative optimism for infection were positively correlated with trust in sources.

## Discussion

Our findings corroborate earlier work on comparative/unrealistic optimism and self-/illusory superiority for health in general (Hoorens, 1994; Hoorens & Harris, 1998; Weinstein, 1987) and COVID-19 in particular (e.g., Asimakopoulou et al., 2020; Park et al., 2021). Participants generally felt that they were less at risk to get infected with COVID-19 and to suffer severe symptoms in case of an infection, and they also felt that they had in the last month adhered better and would in the next month adhere better than average to the precautions. As in research on other risks (Harris, 1996; Hoorens & Buunk, 1993; Hoorens et al., 2008; Klein & Helweg-Larsen, 2002), comparative optimism was greater for events that participants considered more controllable (infection) than for events that they considered less controllable (severity), and it was greater among participants who perceived more (vs. less) control over both. Although the risk of getting infected and the risk of suffering severe illness after an infection arguably differ on various dimensions other than controllability, it seems that perceived control explains at least part of the difference. The greater comparative optimism for infection in older participants was also consistent with earlier findings (e.g., Dolinski et al., 2020), as was the absence of a meaningful gender difference (e.g.Asimakopoulou et al., 2020; Kuper-Smith et al., 2020; Vieites et al., 2021).

Our findings extend knowledge about self-uniqueness beliefs/biases concerning an infectious disease in various manners. Instead of the expected general egocentric impact bias, we observed an allocentric impact bias. Participants of all ages felt that it was, overall, easier for them to adhere to the rules, and participants of 35 years or older felt that the precautionary rules affected specific domains of their lives less than those of their peers. There was one notable exception: participants under 25 years old felt that the rules affected their lives more than those of their peers. One explanation for the general pattern might be that participants interpreted the general question (and from a given age on also the more specific questions) as probing into their resourcefulness, such that allocentric impact perception reflected self-superiority. Supporting this interpretation, egocentric impact perception was negatively correlated (i.e., allocentric impact perception was positively correlated) with self-superiority and comparative optimism for infection.

The more comparative optimism for infection and the more self-superiority participants showed, the fewer information sources they used. In addition, the more self-superiority and egocentric (less allocentric) impact perception they showed, the less they trusted information sources. Earlier research showed that comparative optimism is associated with lower use of health information (e.g., Zlatev et al., 2010). We showed that this is specifically true for comparative optimism for infection, but not for severity, and that it is also true for other self-uniqueness phenomena.

We also found more information seeking in participants who were high on egocentric impact perception, and higher trust in sources in participants high on comparative optimism for infection. Although these findings are counterintuitive, they can be explained. The more people feel that behavioural precautions are likely to affect them, the more important it may be for them to learn all possible details of the precautions. Seeking information from multiple sources may help them to do so. Comparative optimism for infection may render the information that various sources provide less threatening than it otherwise would be, and thus enhance people’s openness to it.

Of course, we do not claim that self-uniqueness is the sole correlate of trust in information or its sources. For example, one study showed that complex language in public health messages may in some individuals (those believing in conspiracy theories) negatively affect trust (Schnepf et al., 2021). However, we did show that self-uniqueness is associated with individual differences in use of and trust in information sources.

Our findings furthermore extend earlier research by showing the importance, in the case of a contagious illness, to measure comparative/unrealistic optimism for infection and for severity separately. Different levels of comparative/unrealistic optimism occurred for infection and severity. In addition, individual differences in comparative optimism and self-superiority were differently related to vulnerability and personal experience with COVID-19. Being older or having health-related risk factors was associated with lower comparative optimism for severity, but higher comparative optimism for infection. Experience was associated with lower comparative optimism for infection and higher comparative optimism for severity. In addition, comparative optimism for infection and for severity were differently associated with self-superiority, use of information sources, and trust in these sources. Thus, studies that measure relative likelihoods of getting infected only (e.g., Attema et al., 2021; Dolinski et al., 2020) may not only lead to distorted estimates of the extent to which people are comparatively optimistic about infectious diseases, but also obscure the relationship between comparative optimism, its determinants and consequences, and other self-uniqueness beliefs.

### The kernel of truth and the bias in self-uniqueness

We found indications that individual differences in comparative optimism, self-superiority, and egocentric and allocentric impact perception had a kernel of truth. Participants with health-related risk factors were less comparatively optimistic (more pessimistic) about the severity of their symptoms should they get infected than participants without such risk factors. Greater comparative optimism concerning the severity of COVID-19 after having suffered from the disease (vs. not having suffered from it yet) may also have been justified. Patients who before early May 2020 had recovered enough to participate must indeed have suffered relatively mild symptoms.

However, three (sets of) findings cannot reflect ‘objective’ differences. First, we observed age differences even though participants compared themselves to people of their own age. Second, we found lower comparative optimism for infection with COVID-19 in participants with (vs. without) personal experience with it. Lower comparative optimism for an event after having experienced it is consistent with earlier research (Burger & Palmer, 1992; Helweg-Larsen et al., 2011; Weinstein, 1987), but it is surprising in the case of a disease that entails (temporary) immunity. Third, the greater self-superiority among participants with (vs. without) risk factors occurred because they claimed to adhere better to the rules than participants without risk factors (which intuitively makes sense) *but also* because they claimed more than participants without risk factors that others to some extent failed to adhere to them (which cannot have any objective basis).

### Strengths and Limitations

Measuring different self-uniqueness phenomena allowed us to examine relationships between them. Moreover, we went beyond a demonstration of self-uniqueness by examining its relationship with people’s stance towards information sources. Our multi-item measure of comparative optimism allowed us to show that comparative optimism for infection and severity are distinct phenomena. It thus points at the desirability of examining comparative optimism concerning various aspects of risks. We are not the first to separately measure comparative optimism for the severity of illness should one get infected, but earlier studies that also did so used fewer and arguably more ambiguous items than we did; for example, by asking participants how likely they were to experience ‘serious’ symptoms and leaving it up to them which symptoms counted as serious, or broadly defining ‘serious’ as any symptom that entailed hospitalization (cf. Vieites et al., 2021; Wise et al., 2020).

Our large sample size ensured that the sample included individuals with different levels of experience with and risk factors for COVID-19, which allowed us to examine their role in self-uniqueness. Another positive consequence was that small effects could be identified. That was particularly the case for the correlations between self-uniqueness beliefs and stances towards information sources.

However, the smallness of correlations is at the same time a limitation, particularly when they are evaluated with the traditional ‘variance explained’ standard. However, even small effects may be consequential (Prentice & Miller, 1992), particularly when their consequences accumulate (Funder & Ozer, 2019). Still, the replicability of the relationship between self-uniqueness beliefs and stance towards information sources will be an issue to evaluate in follow-up research.

Our sample was diverse in terms of age, but like in many online health surveys, predominantly White and female (Bethlehem, 2010). The large sample size allowed meaningful comparisons across genders, but not across ethnicities. Caution is in order while generalizing to other groups. However, it should be noted that most people in Dutch-speaking Belgium and The Netherlands identify as white. Thus, a sample like ours implies not such a strong selection effect as it would in many other countries.

We examined differences in self-uniqueness as a function of participants’ personal experience with COVID-19, but not as a function of their vicarious experience, e.g., through relatives, friends, and acquaintances who had been infected, had fallen ill, and has perhaps died of the disease. It should be noted that even personal experience played a meaningful role in comparative optimism only. Moreover, earlier research found no systematic effect of vicarious experience on comparative optimism (Kuper-Smith et al., 2021). However, future research may compare the role of personal and vicarious experience in self-uniqueness phenomena.

Our study was cross-sectional and thus did not allow causal conclusions. Yet, some relationships are arguably more likely than others. For example, it is more likely that risk factors affect self-uniqueness than that the latter contributes to risk factors. Still, the observed associations should be considered with caution. A related issue that the age effects may reflect a developmental effect, a cohort effect, or both.

Our comparative optimism and egocentric impact measures asked participants to give comparative ratings rather than absolute ones for them and the average other. Although we had good reasons to do so (see Methods section), the downside was that we could not determine if observed effects could be decomposed as subgroups having a more positive view of their own risks and behaviors and the precautions’ impact on the self, a more negative view concerning the risks, behaviors, and impact on/for others, or both. Yet, our self-superiority findings suggest that the third option is the more likely one.

### Implications for public health communication

We limit ourselves to two take-home messages: that messages should avoid activating self-uniqueness, and that a tailor-made approach targeting groups differing in self-uniqueness may be called for rather than a ‘one size fits all’ approach.

#### Counteracting self-uniqueness

Public health communication may be more effective if it focuses on the severity of a disease rather than its prevalence, given that people show less comparative optimism for the latter than the former (Blanton et al., 2013). However, people’s resistance against behavioural precautions may also derive from the perception that they comply better than others (self-superiority bias). It is therefore important to raise awareness that feelings of doing better are normal but often erroneous.

Public health messages should at the very least mention self-uniqueness and explain why it is misleading, if only by raising awareness that it is widely shared. Recent work has shown that it is possible to reduce comparative optimism through media messages about the extent to which other people follow medical recommendations Dolinski et al., 2021). Thus, reducing self-uniqueness is not impossible.

#### Tailoring messages to audiences

Societal groups differ in self-uniqueness, and public health messages should take those differences into account. For example, we found more comparative optimism and self-superiority in older people, but more egocentric impact perception in younger ones. These differences suggest that messages for different groups should consider different self-uniqueness phenomena to different extents.

Young people seem to believe that behavioural precautions affect them more than their average peer. Besides being stressful, this relative deprivation may entail a lower willingness to adhere to the rules. In older groups, the main problems are comparative optimism for infection and self-superiority. It may thus be useful to raise awareness among young people of what the rules mean for their peers, and among older people of their relative risk of infection, and of how dutifully their peers adhere to the rules.

## Conclusion

People show self-uniqueness beliefs concerning COVID-19. It may be a good idea for public health communicators to tailor their messages such that they minimize comparative optimism, self-superiority, and egocentric impact perception. While doing so, they should consider the self-uniqueness phenomena that are dominant in their target audience.

## Footnotes

N = 2483 partially filled out the survey. We used complete records only as the demographic questions were at the end.

After the data collection in other languages had started, we discovered that the (absolute) labels differed from the (comparative) labels in earlier comparative optimism research. We used the Dutch sampling, which began later, to test if that affected the results. As Supplemental Materials show, that was not the case. We present results for pooled data; using label as a covariate did not alter them.
